# A Zinc Cluster Transcription Factor Contributes to the Intrinsic Fluconazole Resistance of Candida auris

**DOI:** 10.1128/mSphere.00279-20

**Published:** 2020-04-22

**Authors:** Eva-Maria Mayr, Bernardo Ramírez-Zavala, Ines Krüger, Joachim Morschhäuser

**Affiliations:** a Institut für Molekulare Infektionsbiologie, Universität Würzburg, Würzburg, Germany; University of Georgia

**Keywords:** *Candida auris*, fluconazole resistance, transcription factor

## Abstract

The recently emerged pathogenic yeast Candida auris is a major concern for human health, because it is easily transmissible, difficult to eradicate from hospitals, and highly drug resistant. Most C. auris isolates are resistant to the widely used antifungal drug fluconazole due to mutations in the target enzyme Erg11 and high activity of efflux pumps, such as Cdr1. In the well-studied, distantly related yeast Candida albicans, overexpression of drug efflux pumps also is a major mechanism of acquired fluconazole resistance and caused by gain-of-function mutations in the zinc cluster transcription factors Mrr1 and Tac1. In this study, we investigated a possible involvement of related transcription factors in efflux pump expression and fluconazole resistance of C. auris. The C. auris genome contains three genes encoding Mrr1 homologs and two genes encoding Tac1 homologs, and we generated deletion mutants lacking these genes in two fluconazole-resistant strains from clade III and clade IV. Deletion of *TAC1b* decreased the resistance to fluconazole and voriconazole in both strain backgrounds, demonstrating that the encoded transcription factor contributes to azole resistance in C. auris strains from different clades. *CDR1* expression was not or only minimally affected in the mutants, indicating that Tac1b can confer increased azole resistance by a *CDR1*-independent mechanism.

**IMPORTANCE** Candida auris is a recently emerged pathogenic yeast that within a few years after its initial description has spread all over the globe. C. auris is a major concern for human health, because it can cause life-threatening systemic infections, is easily transmissible, and is difficult to eradicate from hospital environments. Furthermore, C. auris is highly drug resistant, especially against the widely used antifungal drug fluconazole. Mutations in the drug target and high activity of efflux pumps are associated with azole resistance, but it is not known how drug resistance genes are regulated in C. auris. We have investigated the potential role of several candidate transcriptional regulators in the intrinsic fluconazole resistance of C. auris and identified a transcription factor that contributes to the high resistance to fluconazole and voriconazole of two C. auris strains from different genetic clades, thereby providing insight into the molecular basis of drug resistance of this medically important yeast.

## Abstract (precis)

Candida auris is a recently emerged pathogenic yeast that within a few years after its initial description has spread all over the globe. C. auris is a major concern for human health, because it can cause life-threatening systemic infections, is easily transmissible, and is difficult to eradicate from hospital environments. Furthermore, C. auris is highly drug resistant, especially against the widely used antifungal drug fluconazole. Mutations in the drug target and high activity of efflux pumps are associated with azole resistance, but it is not known how drug resistance genes are regulated in C. auris. We have investigated the potential role of several candidate transcriptional regulators in the intrinsic fluconazole resistance of C. auris and identified a transcription factor that contributes to the high resistance to fluconazole and voriconazole of two C. auris strains from different genetic clades, thereby providing insight into the molecular basis of drug resistance of this medically important yeast.

## INTRODUCTION

Candida auris is a recently emerged pathogenic *Candida* species that was described for the first time only a decade ago (1) but has since been isolated with increasing frequency from all over the world (2). Four genetically distinct clades of C. auris have been identified and named according to their geographic origin: South Asian (clade I), East Asian (clade II), South African (clade III), and South American (clade IV) (3). Within each clade, isolates are genetically nearly identical, but strains from different clades differ by tens of thousands of single nucleotide polymorphisms. C. auris is a major health concern, because it persists on skin and hospital surfaces despite disinfection measures, is easily transmitted, and has already caused nosocomial outbreaks (4). Except for strains from clade II, which have been mainly isolated from the ear canal of patients, C. auris can cause systemic infections with high mortality rates. This problem is aggravated by the fact that C. auris is a highly drug-resistant species, and resistance to all classes of antifungal drugs that are available to treat such infections has been reported. Resistance to the most widely used antifungal drug, fluconazole, is especially prominent, with >90% of all isolates being resistant, such that C. auris can be considered an intrinsically fluconazole-resistant species that requires alternative drugs for treatment. Fluconazole resistance is frequently associated with mutations in the target enzyme Erg11, a well-known azole resistance mechanism in other fungal pathogens (3, 5–7). However, *ERG11* mutations alone cannot explain the very high fluconazole resistance levels of many C. auris strains, and high activity of drug efflux pumps has been suggested as an additional mechanism (8).

In the well-studied, distantly related pathogenic yeast Candida albicans, which is normally susceptible to fluconazole but can acquire resistance to the drug under selective pressure, three efflux pumps are known to contribute to azole resistance, the ABC transporters Cdr1 and Cdr2 and the major facilitator Mdr1 (9). Constitutive overexpression of the encoding genes is caused by gain-of-function (GOF) mutations in the zinc cluster transcription factors Tac1 and Mrr1, respectively, and is a major mechanism of acquired azole resistance in C. albicans (10–14). A *CDR1* homolog has been identified in C. auris and shown by targeted gene deletion to mediate azole resistance (15, 16). How the transcription of *CDR1* is regulated in C. auris and how high expression levels resulting in drug resistance are achieved in this species are currently unknown. We hypothesized that homologs of Tac1 and Mrr1 might control expression of *CDR1* and other potential drug efflux pump-encoding genes also in C. auris and be constitutively active to ensure the high fluconazole resistance of many strains. We therefore identified *TAC1* and *MRR1* homologs in the C. auris genome and investigated their possible involvement in azole resistance.

## RESULTS

### Identification of *MRR1* and *TAC1* homologs in C. auris.

Highly complete genome assemblies have recently been published for four C. auris isolates representing each clade of this species (17). Among these isolates, B11243 (clade IV) exhibited the highest level of fluconazole resistance (>256 μg/ml), which cannot be explained solely by the Y132F mutation found in Erg11 of this strain. We therefore chose isolate B11243 to investigate a possible role of Mrr1 and Tac1 homologs in fluconazole resistance of C. auris. A BLAST search of the B11243 genome sequence with the Mrr1 and Tac1 protein sequences of C. albicans strain SC5314 identified three and two predicted proteins, respectively, for which a reciprocal BLAST search yielded Mrr1 and Tac1 as best hits. For simplicity, we designated the corresponding genes *MRR1a* (PSK78296.1), *MRR1b* (PSK79149.1), *MRR1c* (PSK77655.1), *TAC1a* (PSK79380.1), and *TAC1b* (PSK79381.1) in the present study. *MRR1a* encodes a protein of 1,133 amino acids that has 35.4% identity and 54% similarity over its entire length to CaMrr1. *MRR1b* encodes a protein of 1,059 amino acids with 28.8% identity and 47.1% similarity to CaMrr1. *MRR1c* encodes a protein of 851 amino acids with 25.3% identity and 40.5% similarity to CaMrr1. Of the two Tac1 homologs, Tac1a (805 amino acids) has 29% identity and 46.4% similarity to CaTac1, while Tac1b (863 amino acids) has 26.7% identity and 45.4% similarity to CaTac1. *TAC1a* and *TAC1b* are located in tandem in the genome of C. auris, suggesting that they arose by gene duplication; however, the encoded proteins display only low similarity to each other (24.4% identity and 43.6% similarity). All five predicted proteins contain the consensus motif CX_2_CX_6_CX_5–12_CX_2_CX_6–8_C in their N-terminal region, as is typical for zinc cluster transcription factors (18).

### Deletion of *MRR1* and *TAC1* homologs in C. auris strain B11243.

**FIG 1:**
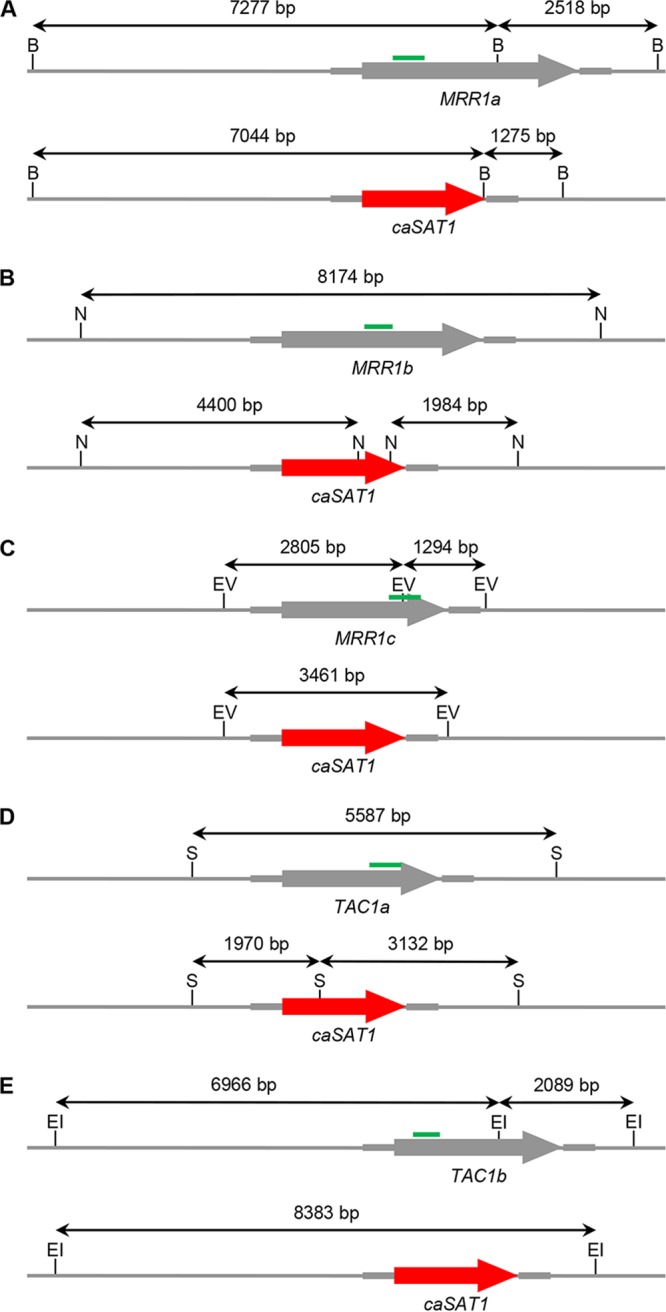
Schematics of the genomic *MRR1a* (A), *MRR1b* (B), *MRR1c* (C), *TAC1a* (D), and *TAC1b* (E) loci in the wild-type strain B11243 (top panels) and after replacement of the coding sequences by the *caSAT1* marker via homologous recombination (bottom panels). Diagnostic restriction sites used to analyze transformants are indicated: B, BamHI; N, NdeI; EV, EcoRV; S, SalI; EI, EcoRI. The coding regions of the target genes are indicated by the gray arrows, and the *caSAT1* marker is indicated by the red arrows. The gray lines represent the flanking sequences; the thicker parts were cloned in the deletion cassettes and also used as upstream and downstream probes in Southern hybridizations. The predicted sizes of hybridizing fragments in the parental strain and deletion mutants are given. The green bars indicate the parts of the coding sequences that were used as probes to confirm the absence of the target genes in deletion mutants.

To investigate if the Mrr1 and Tac1 homologs are important for the high fluconazole resistance of C. auris strain B11243, we generated deletion mutants lacking the coding sequences of the corresponding genes. In contrast to C. albicans, C. auris is a haploid species, so that null mutants can be obtained in a single gene replacement step. We constructed deletion cassettes in which the *caSAT1* selection marker (19), which confers resistance to nourseothricin and has been used successfully by other researchers for the genetic manipulation of C. auris (16), was flanked by ca. 0.5 kb of the upstream and downstream sequences of the target genes. Nourseothricin-resistant clones that were obtained after transformation with the deletion cassettes were then analyzed by Southern hybridization of genomic DNA that was digested with suitable restriction enzymes (schematics in Fig. 1).

**FIG 2:**
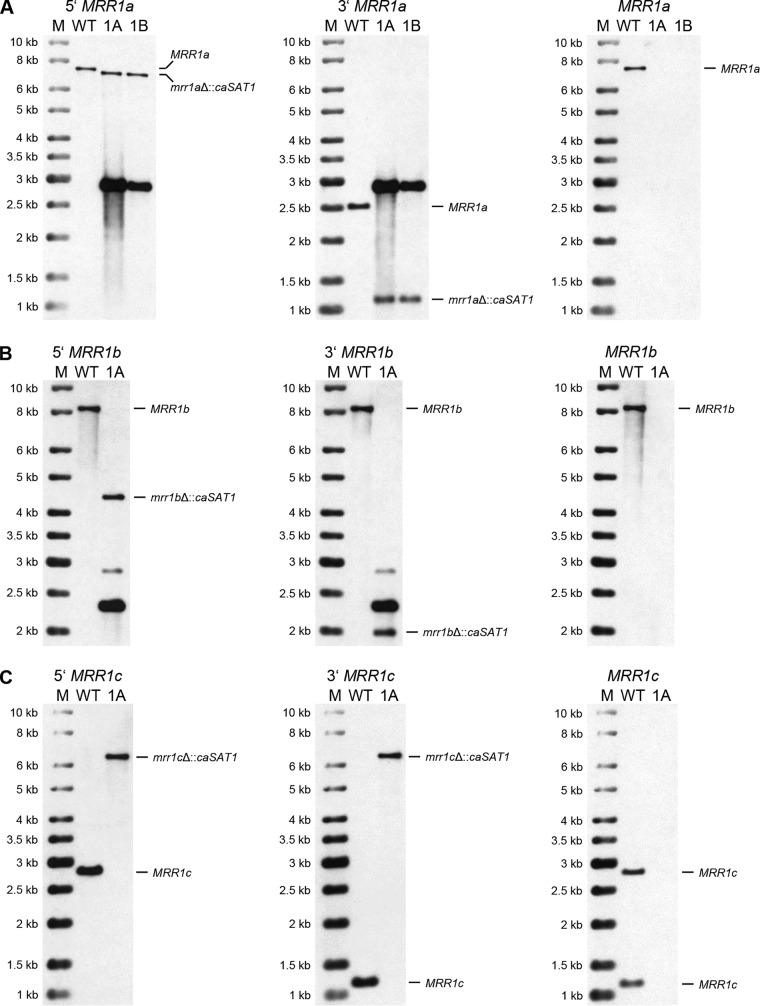
Southern hybridization analysis of the parental strain B11243 (WT) and the *mrr1a*Δ (A), *mrr1b*Δ (B), and *mrr1c*Δ (C) mutants derived from it. Genomic DNA of the strains was digested with appropriate restriction enzymes as shown in Fig. 1 and hybridized with upstream probes (left panels), downstream probes (middle panels), and open reading frame (ORF) probes (right panels). Molecular size markers (M) are on the left, and the identity of hybridizing fragments is given on the right side of the blots. Names of the mutants are abbreviated; for example, 1A and 1B in panel A indicate the *mrr1a*Δ mutants AR0931MRR1aM1A and AR0931MRR1aM1B, respectively.

Among 26 tested clones that were transformed with the *MRR1a* deletion cassette, only four had lost the wild-type BamHI fragments and contained the expected new fragments after hybridization with the upstream and downstream flanking sequences, whereas the other transformants had ectopically integrated the deletion cassette. The correct transformants contained an additional hybridizing fragment of 2.9 kb that produced a strong signal with the probes and corresponded to the size of the *MRR1a* deletion cassette (which contained a single BamHI site), indicative of tandem integration of multiple copies of the cassette. This hybridizing fragment was also present in most of the clones with ectopic integrations. Rehybridization of the blot with a probe from the *MRR1a* coding sequence confirmed the deletion of the target gene in the four correct transformants, and two clones from independent sets of transformants, termed AR0931MRR1aM1A and -B (Fig. 2A), were kept for phenotypic analysis (Table 1 lists all strain names and genotypes).

**TABLE 1:** C. auris strains

Strain	Parent	Genotype	Reference
B11243 (AR#0931)		Clinical isolate	17
AR0931MRR1aM1A and B	B11243	*mrr1a*Δ::*caSAT1*^*a*^	This study
AR0931MRR1bM1A	B11243	*mrr1b*Δ::*caSAT1*^*b*^	This study
AR0931MRR1cM1A	B11243	*mrr1c*Δ::*caSAT1*^*a*^	This study
AR0931TAC1aM1A and B	B11243	*tac1a*Δ::*caSAT1*^*b*^	This study
AR0931TAC1bM1A and B	B11243	*tac1b*Δ::*caSAT1*	This study
AR0931TAC1bM2A	B11243	*tac1b*Δ::*cauSAT1*	This study
B11221 (AR#0383)		Clinical isolate	17
AR0383MRR1aM1A	B11221	*mrr1a*Δ::*caSAT1*	This study
AR0383MRR1bM1A	B11221	*mrr1b*Δ::*caSAT1*	This study
AR0383MRR1cM1A	B11221	*mrr1c*Δ::*caSAT1*	This study
AR0383TAC1aM1A and B	B11221	*tac1a*Δ::*caSAT1*	This study
AR0383TAC1bM1A	B11221	*tac1b*Δ::*caSAT1*	This study

a Strains contain tandem integrations of the deletion cassette.

b Strain contains additional ectopic integrations.

Of seven tested clones that were transformed with the *MRR1b* deletion cassette, only one had lost the wild-type NdeI fragment and contained the expected new fragments after hybridization with the upstream and downstream flanking sequences (Fig. 2B); the other transformants had ectopically integrated multiple copies of the deletion cassette. The positive clone additionally contained a strongly hybridizing 2.3-kb fragment, which could be explained by tandem integration of more than one copy of the deletion cassette. Rehybridization with a probe from the *MRR1b* coding sequence confirmed its deletion in strain AR0931MRR1bM1A (Fig. 2B).

Among five analyzed transformants with the *MRR1c* deletion construct, only one had lost the wild-type EcoRV fragments, while the other clones contained ectopic insertions. The size of the single new hybridizing fragment (6.3 kb) indicated that two copies of the deletion cassette were integrated at the endogenous *MRR1c* locus of the deletion mutant AR0931MRR1cM1A (Fig. 2C).

**FIG 3:**
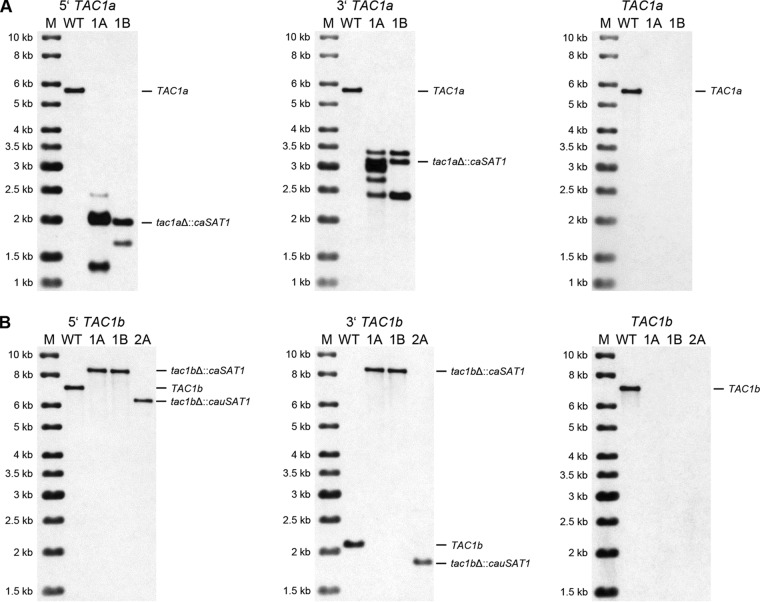
Southern hybridization analysis of the parental strain B11243 (WT) and the *tac1a*Δ (A) and *tac1b*Δ (B) mutants derived from it. See Fig. 2 for explanations. The *tac1b*Δ mutant AR0931TAC1bM2A (2A) has a different hybridization pattern because the *cauSAT1* marker contains an internal EcoRI site that is not present in the *caSAT1* marker used to generate AR0931TAC1bM1A (1A) and AR0931TAC1bM1B (1B).

For *TAC1a*, we analyzed 36 clones and identified two independent transformants in which the target gene was deleted. In addition to the predicted new SalI fragments, several extra fragments hybridized with the upstream and downstream flanking sequences in these transformants (Fig. 3A), suggestive of additional integration events. Rehybridization with a probe from the *TAC1a* coding region confirmed the deletion of *TAC1a* in strains AR0931TAC1aM1A and -B.

Our initial efforts to delete *TAC1b* in C. auris strain B11243 were unsuccessful, as none of the first 48 analyzed transformants displayed the desired allelic replacement. Since the majority of our transformants in the previous experiments contained multiple and often ectopically integrated copies of the deletion cassettes (see above), we hypothesized that the *caSAT1* marker might not be well expressed in C. auris and the nourseothricin concentration used in our experiments (200 μg/ml) selected for clones with multiple copies. We also observed that B11243 and several other tested C. auris strains were more sensitive to nourseothricin than C. albicans and did not grow on plates containing 10 μg/ml of the antibiotic, on which C. albicans can still grow (19). We therefore lowered the nourseothricin concentration in the selection plates and obtained five mutants with the correct hybridization pattern from plates with 50 μg/ml nourseothricin (out of 24 tested transformants) and two additional correct deletion mutants out of 12 tested clones from plates with 100 μg/ml nourseothricin. Two independent *tac1b*Δ mutants, AR0931TAC1bM1A and -B (Fig. 3B), were kept for phenotypic analysis. In parallel, we replaced the C. albicans *ACT1* promoter in the *caSAT1* marker by the upstream region of the C. auris *ACT1* gene (see Materials and Methods) to improve expression of the so-generated modified nourseothricin resistance marker (termed *cauSAT1* to distinguish it from *caSAT1*). This did not increase the frequency of correct transformants, but we obtained an additional mutant (out of 24 tested transformants) from a plate with 200 μg/ml nourseothricin. In this mutant (AR0931TAC1bM2A), *TAC1b* was also replaced by a single copy of the marker (Fig. 3B).

### *TAC1b* contributes to azole resistance in C. auris strain B11243.

To investigate if deletion of any of the *MRR1* and *TAC1* homologs resulted in increased susceptibility of the mutants to fluconazole, we compared the fluconazole MICs for the parental strain B11243 and the deletion mutants. We confirmed the reported high fluconazole resistance of isolate B11243 (MIC, >256 μg/ml). All mutants lacking *MRR1a*, *MRR1b*, *MRR1c*, or *TAC1a* displayed the same high resistance, indicating that the encoded transcription factors did not detectably contribute to fluconazole resistance in strain B11243. In contrast, the fluconazole MIC for all three independently generated *tac1b*Δ mutants was reduced to between 128 μg/ml and 256 μg/ml, i.e., at least 2- to 4-fold (Table 2). Strain B11243 is also highly resistant to voriconazole, with a reported MIC of 8 μg/ml (17). In our assays, the MIC of voriconazole for B11243 was 4 μg/ml and no decrease was observed after deletion of *MRR1a*, *MRR1b*, *MRR1c*, and *TAC1a* (the MIC was even minimally increased for the *tac1a*Δ mutants). In contrast, the MIC of voriconazole was reduced to between 0.5 μg/ml and 1 μg/ml, i.e., 4- to 8-fold, for all three *tac1b*Δ mutants of this strain (Table 2). These results demonstrate that Tac1b contributes to fluconazole and voriconazole resistance in strain B11243, although the mutants retained high resistance levels.

**TABLE 2:** MICs of fluconazole and voriconazole for C. auris strains

Strain	Genotype	MIC (μg/ml) of drug:	
		Fluconazole	Voriconazole
B11243 (AR#0931)	Wild type	>256	4
AR0931MRR1aM1A	*mrr1a*Δ	>256	4
AR0931MRR1aM1B	*mrr1a*Δ	>256	4
AR0931MRR1bM1A	*mrr1b*Δ	>256	4
AR0931MRR1cM1A	*mrr1c*Δ	>256	4
AR0931TAC1aM1A	*tac1a*Δ	>256	4–8
AR0931TAC1aM1B	*tac1a*Δ	>256	4–8
AR0931TAC1bM1A	*tac1b*Δ	128–256	0.5–1
AR0931TAC1bM1B	*tac1b*Δ	128–256	0.5–1
AR0931TAC1bM2A	*tac1b*Δ	128–256	0.5–1
B11221 (AR#0383)	Wild type	256	1–2
AR0383MRR1aM1A	*mrr1a*Δ	128	0.5–1
AR0383MRR1bM1A	*mrr1b*Δ	256	1–2
AR0383MRR1cM1A	*mrr1c*Δ	256	1–2
AR0383TAC1aM1A	*tac1a*Δ	256	1–2
AR0383TAC1aM1B	*tac1a*Δ	256	1–2
AR0383TAC1bM1A	*tac1b*Δ	128	0.5–1

### Deletion of *MRR1* and *TAC1* homologs in C. auris strain B11221.

**FIG 4:**
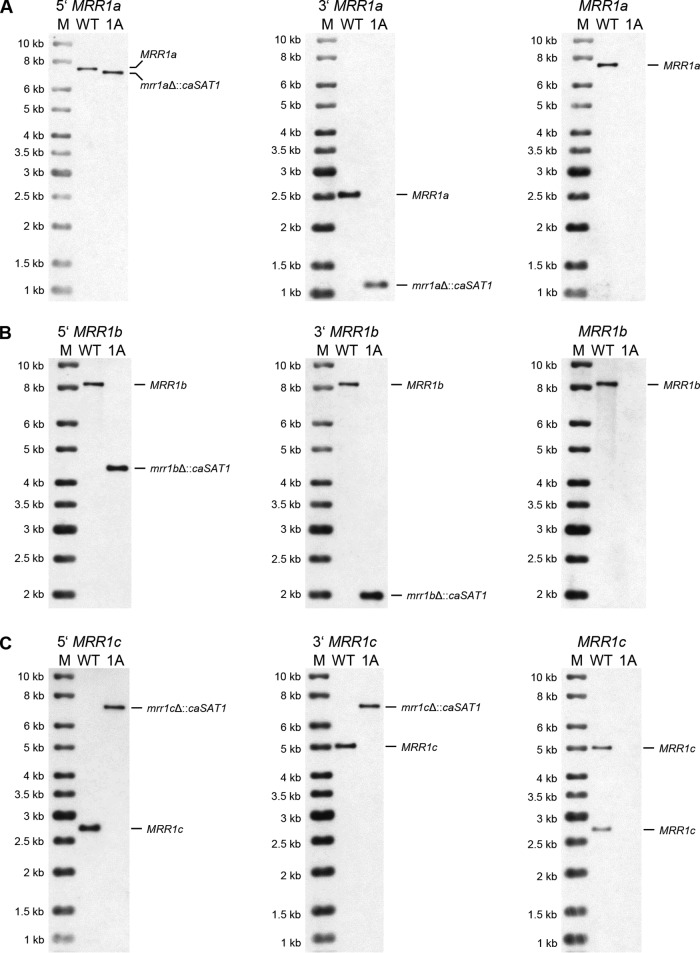
Southern hybridization analysis of the parental strain B11221 (WT) and the *mrr1a*Δ (A), *mrr1b*Δ (B), and *mrr1c*Δ (C) mutants derived from it. See Fig. 2 for explanations.

**FIG 5:**
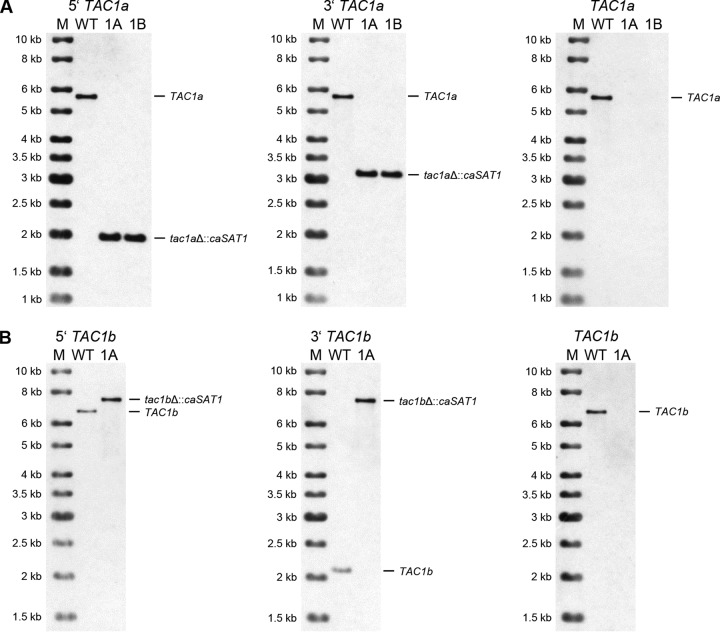
Southern hybridization analysis of the parental strain B11221 (WT) and the *tac1a*Δ (A) and *tac1b*Δ (B) mutants derived from it. See Fig. 2 for explanations.

Since the MIC of fluconazole for strain B11243 was above the highest tested concentration (256 μg/ml), a minor contribution of *TAC1a* and the *MRR1* homologs of C. auris to the high fluconazole resistance of this strain might not have been detected in our experiments. We therefore chose an additional fluconazole-resistant C. auris strain (B11221, clade III) with a reported fluconazole MIC of 64 μg/ml (17) to generate a separate set of deletion mutants. B11221 also contains an Erg11 mutation (F126L) that is supposed to contribute to its fluconazole resistance but that cannot fully explain it (17). The Mrr1 and Tac1 homologs of strain B11221 are highly similar to their counterparts in strain B11243, with identities of 98.3% for Mrr1a (PIS54262.1), 97.8 for Mrr1b (PIS53339.1), 99.9% for Mrr1c (PIS50876.1), 99.3 for Tac1a (PIS49946.1), and 97.9% for Tac1b (PIS49945.1). With this strain, we faced the same problem of unspecific integration of the deletion cassettes, which may have been exacerbated by the fact that the flanking sequences were derived from strain B11243 and are not identical to those in strain B11221. Nevertheless, we obtained one *mrr1a*Δ mutant (AR0383MRR1aM1A) out of 60 tested transformants, two *mrr1b*Δ mutants out of 36 tested transformants (only one, AR0383MRR1bM1A, was kept, because they were recovered from the same plate), and two independent *tac1a*Δ mutants (AR0383TAC1aM1A and -B) out of 36 tested transformants after selection with 50 μg/ml nourseothricin. In all these mutants, the target gene was correctly replaced by a single copy of the *caSAT1* marker (Fig. 4A and B and Fig. 5A). We did not obtain *mrr1c*Δ and *tac1b*Δ mutants with the previously used deletion cassettes (48 clones were tested in each case) and therefore tried a split marker approach in an effort to increase the frequency of homologous recombination (20, 21) (see Materials and Methods). Although this also did not improve the efficiency of gene deletion, we obtained one mutant for each gene (out of 36 tested clones in each case). The *mrr1c*Δ mutant AR0383MRR1cM1A (Fig. 4C) exhibited the expected hybridization pattern (the EcoRV fragments on which *MRR1c* is located in strain B11221 are different from those in strain B11243; the 2,718-bp and 4,973-bp fragments hybridizing with the upstream and downstream sequences, respectively, in the wild-type parent were predicted to be replaced by a single 7,053-bp fragment hybridizing with both probes in correct mutants). The EcoRI restriction pattern at the *TAC1b* locus is also different in B11221 compared to B11243, and the 6,581-bp upstream and the 2,105-bp downstream fragment were predicted to be replaced by a single 8,014-bp fragment after insertion of the *caSAT1* marker. In the *tac1b*Δ mutant AR0383TAC1bM1A, the new fragment that hybridized with both the upstream and downstream probes was slightly smaller than expected (Fig. 5B), indicating that the allelic exchange was unprecise, most likely because of sequence differences in the flanking homology regions. Rehybridization with probes from the coding sequences confirmed the deletion of all five target genes also in the mutants derived from strain B11221 (Fig. 4 and 5).

**FIG 6:**
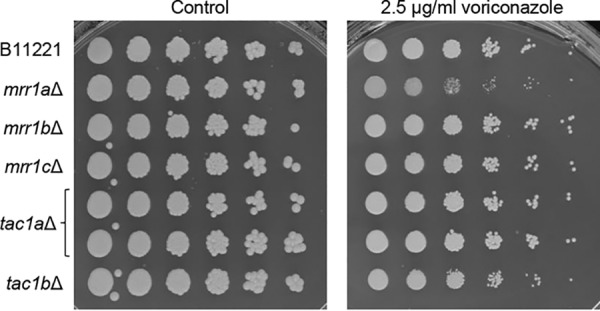
Voriconazole sensitivity of strain B11221 and deletion mutants. Serial dilutions of the indicated strains were spotted on SD agar plates without (control) or with voriconazole and grown for 4 days at 37°C.

The fluconazole and voriconazole MICs for strain B11221 were somewhat higher in our assays than previously reported (256 μg/ml instead of 64 μg/ml for fluconazole, and 1 μg/ml to 2 μg/ml instead of 0.5 μg/ml for voriconazole) (17). As for strain B11243, deletion of *MRR1b*, *MRR1c*, and *TAC1a* in strain B11221 did not alter the MICs of the two drugs, but the *tac1b*Δ mutant, and in this case also the *mrr1a*Δ mutant, displayed a 2-fold-reduced resistance to fluconazole and voriconazole (Table 2). The reduced voriconazole resistance of the *mrr1a*Δ and *tac1b*Δ mutants was also observed in a dilution spot assay on agar plates (Fig. 6). In summary, Tac1b contributes to the azole resistance of C. auris strains from both clade III and clade IV, while a minor contribution of Mrr1a to azole resistance was observed only in the clade III strain B11221.

### *CDR1* expression in *tac1a*Δ and *tac1b*Δ mutants.

**FIG 7:**
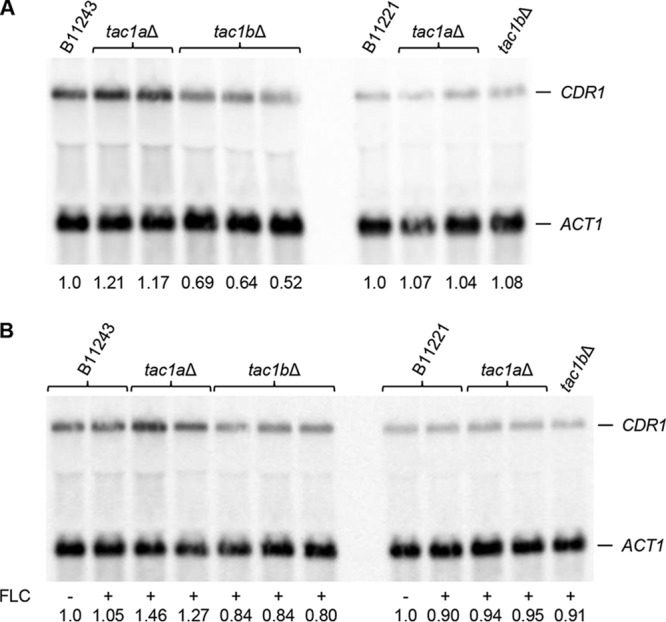
*CDR1* expression in the parental strains B11243 and B11221 and deletion mutants. Strains were grown to log phase without (A) or with (B) fluconazole (FLC) exposure and analyzed by Northern hybridization with *CDR1*- and *ACT1*-specific probes. The identities of the mRNAs are indicated. Signals were quantified and normalized to *ACT1* signals for each strain. The signals of the wild-type strains grown in the absence of fluconazole were set to 1, and the normalized *CDR1* expression values for each strain are given below the blots. The wild-type samples from panel A were included in panel B to compare *CDR1* expression levels in the absence (−) and presence (+) of fluconazole (FLC).

In C. albicans, Tac1 is not required for basal *CDR1* expression levels, but hyperactive forms of Tac1 cause constitutive *CDR1* overexpression (12, 22). We therefore tested if the Tac1 homologs Tac1a and Tac1b regulate *CDR1* expression in C. auris. Northern hybridization analysis showed that *CDR1* transcript levels were slightly reduced in the *tac1b*Δ mutants of strain B11243 but not in the *tac1b*Δ mutant derived from strain B11221 and not in any of the *tac1a*Δ mutants (Fig. 7A). We considered the possibility that Tac1b might upregulate *CDR1* expression in response to fluconazole and thereby promote increased drug resistance. However, *CDR1* mRNA levels were not increased in wild-type cells in the presence of fluconazole and were not or only minimally affected by the deletion of *TAC1a* and *TAC1b* (Fig. 7B). Therefore, the contribution of Tac1b to azole resistance in strains B11243 and B11221 seems to involve other, *CDR1*-independent mechanisms.

## DISCUSSION

The efflux pump encoded by *CDR1* has recently been found to play a major role in the fluconazole resistance of C. auris. *CDR1* deletion decreased the MIC by 4-fold in a fluconazole-susceptible isolate and by 8-fold and 64-fold in two different fluconazole-resistant isolates, all from clade I (15, 16). Since activating mutations in the transcription factor Tac1 are responsible for *CDR1* overexpression in C. albicans and a major cause of azole resistance in this species, we hypothesized that a Tac1 homolog might be constitutively active in C. auris and confer fluconazole resistance by promoting high *CDR1* expression levels. However, deletion of *TAC1a*, one of two *CaTAC1* homologs in C. auris, in two different strains did not affect fluconazole resistance, and deletion of *TAC1b* only mildly reduced their high fluconazole MICs. Furthermore, the Tac1 homologs did not or only minimally contribute to *CDR1* expression in the presence of fluconazole under the conditions used in our experiments. Other transcription factors may therefore be more important for *CDR1* expression in C. auris. We also considered the possibility that *TAC1a* and *TAC1b*, which are located in tandem in the C. auris genome, might have partially redundant roles. Unfortunately, our efforts to construct *tac1a*Δ *tac1b*Δ double mutants of strains B11243 and B11221 were unsuccessful. It is possible that *CDR1* is less critical for azole resistance in the two strains from clades III and IV investigated in our study than in the clade I strains studied previously, in which it may be more strongly expressed, possibly due to activating mutations in Tac1a or Tac1b. Nevertheless, Tac1b contributed to azole resistance in strains B11243 and B11221, even if this did not involve *CDR1* upregulation.

Activating mutations in the transcription factor Mrr1 are another common cause of fluconazole resistance in C. albicans, which is partly mediated by the constitutive overexpression of the efflux pump-encoding gene *MDR1*. C. auris possesses three Mrr1 homologs, but we did not find clear evidence for their involvement in the fluconazole resistance of the two C. auris isolates investigated in our present study. A possible exception is Mrr1a, as *MRR1a* deletion in strain B11221 resulted in a slightly increased susceptibility to fluconazole and voriconazole. A caveat here is that only one *mrr1a*Δ mutant was obtained from this parental strain, and *MRR1a* deletion did not detectably affect azole resistance in strain B11243. We therefore cannot exclude the possibility that the increased azole susceptibility of the *mrr1a*Δ mutant derived from strain B11221 was caused by an unspecific mutation during the construction of the strain.

Since C. auris is a haploid species, we anticipated that the generation of specific gene deletion mutants would be straightforward, as also inferred from the successful construction of C. auris mutants by other researchers (15, 16, 23–26). Unexpectedly, the vast majority of our transformants had unspecifically inserted the five different gene deletion cassettes at ectopic sites in the genome instead of integrating them at the target locus, suggesting that homologous recombination is much less efficient in C. auris than in C. albicans. Ectopic integration apparently was not such a significant problem in studies by other researchers who generated C. auris gene deletion mutants. Grahl et al. (25) used longer flanking sequences (ca. 1 kb) and a different nourseothricin resistance marker (*NAT1*) to delete the *CAT1* gene in a C. auris clade I strain. Of 10 tested transformants, five contained the desired gene replacement. This was slightly improved (7/10) when CRISPR-Cas9 was used to introduce a double-strand break at the genomic target locus. Day et al. (24) also used the *NAT1* marker, but short flanking homology regions (100 bp), to delete the *HOG1* gene in a C. auris strain from clade I. Using this strategy, they found that *HOG1* was accurately deleted in 30% of the nourseothricin-resistant transformants, suggesting that it is not necessary to include long fragments of sequence homology to achieve targeted integration of a selection marker. Kim et al. (15) applied the CRISPR-Cas9 technology to replace the *HSP90* promoter by a doxycycline-repressible promoter and to delete *CDR1* in a clade I strain, using a hygromycin resistance marker or the *NAT1* marker, respectively, for the selection of transformants. However, they did not report the frequency of specific integration events. Rybak et al. (16) also used a CRISPR-Cas9 system in combination with a replacement cassette containing the *caSAT1* selection marker and short (50-bp) flanking homology regions to delete *CDR1* and *MDR1* in two clade I isolates but did not report the frequency of specific gene replacement. Integration specificity was also not detailed in two very recent studies in which the *NAT1* marker and long flanking regions were used for gene deletions in C. auris (23, 26). The reason for the low frequency of specific marker integration into the target locus in our experiments is not evident. Nourseothricin resistance was used for the selection of transformants in all six previous studies, and Rybak et al. (16) also used the same nourseothricin resistance gene (*caSAT1*) as we did. The use of electroporation for transformation of C. auris also does not seem to be the problem, because electroporation was also used in the studies by Grahl et al., Kim et al., Rybak et al., and Iyer et al. (15, 16, 25, 26). It is possible that homologous recombination is less efficient in strains from clade III and clade IV, used to generate mutants in our present study, as opposed to clade I strains, which were the parents of the mutants generated in the six previous studies (15, 16, 23–26). Alternatively, the frequency of specific marker integration may vary considerably depending on the target locus.

Another interesting observation in our experiments was that nourseothricin-resistant transformants often contained multiple copies of the deletion cassette (we note that such multiple integration events would not be detected by the diagnostic PCR methods employed by most researchers to confirm gene replacements). While this might be related to the mechanism used by C. auris to integrate exogenously supplied DNA into the genome, it also suggested that multiple copies of the resistance marker were required for growth on the selection plates. We considered the possibility that the *caSAT1* marker was not as efficiently expressed in C. auris as in C. albicans and conferred lower levels of resistance. Yet, after replacement of the *CaACT1* promoter in the selection marker by the *ACT1* promoter from C. auris, many transformants with the *TAC1b* deletion cassette still contained multiple integrations. We found that wild-type C. auris strains were more susceptible to nourseothricin than wild-type C. albicans, which may have favored growth of transformants containing more than one copy of the resistance marker immediately after plating on selective medium if the marker was not yet efficiently expressed. We therefore lowered the nourseothricin concentration used for selection of transformants from 200 μg/ml to 50 μg/ml in subsequent experiments. Although we still observed multiple ectopic integration events in many transformants, all correct gene deletion mutants selected with the lower nourseothricin concentration (the *tac1b*Δ mutants of strain B11243 and all deletion mutants of strain B11221) contained only a single copy of the selection marker instead of the tandem integrations that had occurred in the previously obtained mutants (*mrr1a*Δ, *mrr1b*Δ, and *mrr1c*Δ mutants of strain B11243). In any case, and from a practical perspective, our results argue that a lower nourseothricin concentration is sufficient for the selection of C. auris transformants when using this rather expensive reagent, which considerably reduces the costs of experiments.

In conclusion, our study has provided novel insights into the molecular basis of drug resistance of the recently emerged pathogenic yeast C. auris, which is a major concern for human health. Our work also provides useful information about the genetic manipulation of C. auris that should be valuable for the increasing community of researchers studying this fungus.

## MATERIALS AND METHODS

### Strains and growth conditions.

The C. auris strains used in this study are listed in Table 1. The clinical isolates B11243 and B11221 were obtained from the Centers for Disease Control and Prevention (CDC AR Bank numbers 0931 and 0383, respectively). All strains were stored as frozen stocks with 17.2% glycerol at −80°C and subcultured on YPD agar plates (10 g yeast extract, 20 g peptone, 20 g glucose, 15 g agar per liter) at 30°C. Strains were routinely grown in YPD liquid medium at 30°C in a shaking incubator.

### Plasmid constructions.

To generate deletion constructs for *MRR1a*, *MRR1b*, *MRR1c*, *TAC1a*, and *TAC1b*, ca. 0.5 kb of the upstream and downstream regions of these genes was PCR amplified from genomic DNA of strain B11243 with the primers listed in Table 3. The upstream fragments were digested at the KpnI and ApaI sites introduced with primers 1 and 2, and the downstream fragments were digested at the SacII and SacI sites introduced with primers 3 and 4, respectively. The upstream and downstream flanking sequences of each target gene were then cloned together with an ApaI-SacII fragment from plasmid pSAT1, which contains the *caSAT1* marker (19), in the KpnI/SacI-digested vector pBluescript II KS, resulting in plasmids pCauMRR1aM1, pCauMRR1bM1, pCauMRR1cM1, pCauTAC1aM1, and pCauTAC1bM1. To obtain a C. auris-adapted *SAT1* marker (*cauSAT1*), a fragment containing 1,059 bp of the upstream region of the C. auris *ACT1* gene was amplified from genomic DNA of strain B11243 with primers CauACT1.01 and CauACT1.02. A part of the *caSAT1* marker, without the *CaACT1* sequences, was amplified with primers SAT9 and SAT10. The PCR products were then used as the templates in a fusion PCR with primers CauACT1.01 and SAT10. The PCR product was digested at the introduced ApaI and SacII sites and substituted for the *caSAT1* marker in pCauTAC1bM1, yielding pCauTAC1bM2.

**TABLE 3:** Oligonucleotide primers

Primer	Sequence (5′–3′)^*a*^
ACT1CauNBF	TCGAGACCTTCAACGTTCCT
ACT1CauNBR	ACGCACATCGACATCACATT
CauACT1.01	ATATGGGCCCGAGTAGTAATTTGTAACGGG
CauACT1.02	CCGAAATTTTCATATTGACTTAATTGAATTCTTCG
CauMRR1a.01	TATAGGTACCTCGTGAACTTCATCATTGTCACACGG
CauMRR1a.02	TATAGGGCCCATACCATTATCAAAGTTTTTCTGGGGAG
CauMRR1a.03	TATACCGCGGTAAGTTTCATACTACGTGAATATACATGCG
CauMRR1a.04	TATAGAGCTCGGTACCTATTTGATTACTTAGCGATACGATCTCC
CauMRR1a_F	GATAACGCTGCACTCGAACA
CauMRR1a_R	AGGGGCCAAAATTGAGTCTT
CauMRR1b.01	TATAGGTACCGAACCGGACAATGTATGCGAACCG
CauMRR1b.02	TATAGGGCCCATTCGCTTTTTGGAGCTTCCGG
CauMRR1b.03	TATACCGCGGATAGCACGGAGTTAGTGACAATTATG
CauMRR1b.04	TATAGAGCTCGGTACCTATCAGAAATAATGGGTATACTGTATCG
CauMRR1b_F	TTACCCATTTGTCCCGGTTA
CauMRR1b_R	CTCCACCATCATACCCATCC
CauMRR1c.01	TATAGGTACCTTCTATTGGCTGATCTTGAACCTTTGTG
CauMRR1c.02	TATAGGGCCCCATTGGGGTGCTGTTGGTGGAAG
CauMRR1c.03	TATACCGCGGTAAGGGATGCTTCGACCTCTG
CauMRR1c.04	TATAGAGCTCGGTACCGTCTGAAAATTCGAGTTCCTCGG
CauMRR1c_F	GCTACTTCCGGCTCTTCCTT
CauMRR1c_R	AGGCACGACGAGCTCAGTAT
CauTAC1a.01	TATAGGTACCGCTTGATCACGCCACAGCAACTTCAC
CauTAC1a.02	TATAGGGCCCGCATACGGCACTTCGGCTGC
CauTAC1a.03	TATACCGCGGTCAGCAATTCTAAAAGAGATACTACAATAC
CauTAC1a.04	TATAGAGCTCGGTACCATAGCTTCTTGAGATTCGAATGAG
CauTAC1a_F	CACCCCACTCGTACACTCCT
CauTAC1a_R	AGTTCATGCACGTTGTCAGC
CauTAC1b.01	TATAGGTACCGATACTACTGCCAGGCTTGACAG
CauTAC1b.02	TATAGGGCCCAGCTTCTTGAGATTCGAATGAGC
CauTAC1b.03	TATACCGCGGTTAACTTTGTAAATAGTATGCTTACCACG
CauTAC1b.04	TATAGAGCTCGGTACCTGTCGAAGACTGTAACAAAGCC
CauTAC1b_F	GGCCGATTCATCCTCAACTA
CauTAC1b_R	CTGTCCACACGCTCAGAAAA
CDR1CauNBF	GCCAGAACCTTCACCAACAT
CDR1CauNBR	ACAACCAGAACCAGGACGAC
SAT9	CAATTAAGTCAATATGAAAATTTCGGTGATCCC
SAT10	TATACCGCGGGACCACCTTTGATTGTAAATAG
SAT1Nrev1	ATGAGACTGTGCGCGACTCC
SAT1Cfor1	GTTCGATGTGCACCTATCCG

a Introduced restriction sites are underlined.

### Strain constructions.

C. auris strains were transformed by electroporation (19) with the gel-purified inserts (KpnI-KpnI fragments) from plasmids pCauMRR1aM1, pCauMRR1bM1, pCauMRR1cM1, pCauTAC1aM1, pCauTAC1bM1, and pCauTAC1bM2. Transformants were initially selected on YPD plates containing 200 μg/ml nourseothricin, but lower concentrations (50 μg/ml and 100 μg/ml) were used in later experiments. For the split-marker approach, the deletion cassettes contained in pCauMRR1cM1 and pCauTAC1bM1 were amplified as two overlapping fragments using primers 1 and SAT1Nrev1 for the 5′ part and primers 4 and SAT1Cfor1 for the 3′ part. The two fragments of each deletion cassette were then used for electroporation. In this case, a functional nourseothricin resistance marker can be regenerated only when the two fragments are joined by recombination between the overlapping sequences in the transformed cells, which might also increase the frequency of integration at the target locus by homologous recombination with the flanking sequences (21). Genomic integration of the deletion cassettes and absence of the target genes were tested by Southern hybridization using the upstream (amplified with primers 1 and 2) and downstream (amplified with primers 3 and 4) flanking sequences as well as parts of the coding sequences (amplified with primers F and R) as probes.

### Isolation of genomic DNA and Southern hybridization.

Genomic DNA from C. auris strains was isolated as described previously for C. albicans (19). The DNA was digested with appropriate restriction enzymes, separated on a 1% agarose gel, transferred by vacuum blotting onto a nylon membrane, and fixed by UV cross-linking. Southern hybridization with enhanced chemiluminescence-labeled probes was performed with the Amersham ECL direct nucleic acid labeling and detection system (GE Healthcare UK Limited, Little Chalfont, Buckinghamshire, United Kingdom) according to the instructions of the manufacturer. A molecular size marker was included in the probes to facilitate size determination of the hybridizing genomic DNA fragments. ECL signals were captured by exposing the membranes to Hyperfilm (GE Healthcare) and digitized with an HP ScanJet 8300 (HP Inc., Palo Alto, CA).

### Northern hybridization analysis.

Overnight cultures of the strains were diluted to an optical density at 600 nm (OD_600_) of 0.4 in fresh YPD medium and grown for 4 h at 30°C. In a separate experiment, to compare *CDR1* expression levels in the various strains in the presence of fluconazole, 50 μg/ml of fluconazole was added to the cultures after 3 h, followed by further incubation for 1 h. Total RNA was extracted using a Quick-RNA fungal/bacterial miniprep kit (Zymo Research, Irvine, CA) according to the manufacturer’s instructions. RNA samples were separated on a 1.2% agarose gel, transferred by capillary blotting onto a nylon membrane, and fixed by UV cross-linking. The blots were simultaneously hybridized with digoxigenin-labeled probes for *CauCDR1* (positions +157 to +615 in the *CDR1* coding sequence, amplified with primers CDR1CauNBF and CDR1CauNBR) and *CauACT1* (positions +374 to +873 in the *ACT1* coding sequence, amplified with primers ACT1CauNBF and ACT1CauNBR). Bound probe was detected with a peroxidase-labeled antidigoxigenin alkaline phosphatase (AP)-conjugate (Roche, Basel, Switzerland). Signals were generated using CSPD (Roche, Basel, Switzerland) as the substrate and captured with the ImageQuant LAS 4000 imaging system (GE Healthcare). Signal intensities were quantified using the image analysis software Fiji (27).

### Azole susceptibility tests.

The azole susceptibilities of the strains were determined by a previously described broth microdilution method (28), with slight modifications. Three to five 2-day-old colonies from a YPD agar plate were suspended in 2 ml of an 0.9% NaCl solution, and 4 μl of the suspension was mixed with 2 ml 2× SD-CSM medium (13.4 g yeast nitrogen base with ammonium sulfate [YNB; MP Biomedicals, Illkirch, France], 40 g glucose, 1.58 g complete supplement medium [CSM; MP Biomedicals]). Stock solutions of fluconazole and voriconazole (Sigma GmbH, Deisenhofen, Germany) were made in water and dimethyl sulfoxide (DMSO), respectively, and 2-fold dilution series were prepared in water, starting from initial concentrations of 512 μg/ml (fluconazole) and 64 μg/ml (voriconazole). One hundred microliters of each drug solution was then mixed with 100 μl of the cell suspension in a 96-well microtiter plate, and the plates were incubated for 48 h at 37°C. The MIC was defined as the drug concentration that abolished or drastically reduced visible growth compared to a drug-free control. For dilution spot assays, YPD overnight cultures of the strains were diluted to an OD_600_ of 2.0. Tenfold dilutions from 10^0^ to 10^−5^ were prepared in a 96-well microtiter plate, and ca. 5 μl of the cell suspensions was transferred with a replicator onto SD agar plates without or with 2.5 μg/ml voriconazole. Plates were incubated for 4 days at 37°C and photographed.
